# Two different mechanisms for the detection of stimulus omission

**DOI:** 10.1038/srep20615

**Published:** 2016-02-05

**Authors:** Shogo Ohmae, Masaki Tanaka

**Affiliations:** 1 Department of Physiology, Hokkaido University School of Medicine, Sapporo 060-8638, Japan; 2 Department of Neuroscience, Baylor College of Medicine, Houston, TX 77030, USA

## Abstract

Although we can detect slight changes in musical rhythm, the underlying neural mechanism remains elusive. Here we show that two distinct mechanisms are automatically selected depending on the speed of the rhythm. When human subjects detected a single omission of isochronous repetitive auditory stimuli, reaction time strongly depended on the stimulus onset asynchrony (SOA) for shorter SOAs (<250 ms), but was almost constant for longer SOAs. For shorter SOAs, subjects were unable to detect stimulus omission when either monaural stimuli or those in different frequencies were randomly presented. In contrast, for longer SOAs, reaction time increased when different tempos were presented simultaneously to different ears. These results suggest that depending on the speed of rhythms, the brain may use either temporal grouping of discrete sounds or temporal prediction of upcoming stimuli to detect the absence of a regular stimulus. Because we also found a similar relationship between reaction time and SOA for both visual and tactile stimuli, dual detection strategies could be generalized to other sensory modalities.

Temporal processing in the range of hundreds of milliseconds is essential for the perception of rhythm. Several lines of evidence suggest that neural processing of rhythms might be different depending on their speed. For example, the temporal binding of periodic events in different sensory modalities consistently fails at temporal frequencies greater than 2–3 Hz, suggesting the existence of a unified mechanism of temporal binding across modalities that operates only at lower frequencies^1^. In addition, as we listen to a series of two alternating auditory tones at different frequencies, they can be perceptually grouped (auditory stream segregation) when each tone is separated by less than several hundreds of milliseconds^2^, which corresponds temporal frequency of >3–5 Hz. Furthermore, a recent model of time perception suggests that the interval between two successive sensory events may be represented as the state-dependent intrinsic patterns of sensory signals when intervals are less than a few hundred milliseconds, but not for longer intervals^3^,^4^. Finally, the event-related potentials to the omission of isochronous repetitive stimuli have different properties depending on the interstimulus intervals with the phase transition at 4–5 Hz^5^,^6^.

Our recent study in non-human primates also supported the hypothesis of different neural mechanisms of temporal processing. We found that monkeys could detect single omission of isochronously presented repetitive audiovisual stimuli (‘missing oddball’ paradigm) for the stimulus onset asynchronies (SOAs) of 100–800 ms^7^. However, we also found that neurons in the cerebellar dentate nucleus exhibited significant firing modulation only for SOAs >200 ms^7^ and that inactivation of the recording sites was effective only in this range^7^, suggesting that this part of the cerebellum might be specifically involved in the detection of stimulus omission for SOAs longer than several hundreds of milliseconds.

Encouraged by these previous observations, we attempted to characterize the different temporal processing to gain insight into the underlying neural mechanisms. To this end, we examined the performance of normal human subjects responding to a single omission of isochronous repetitive stimuli. We primarily used a sequence of auditory stimuli because, unlike monkeys, humans are well capable of entraining auditory rhythms^8^, while the previous studies also showed that humans can reliably detect the omission of visual stimulus sequence^9^,^10^. Our results suggest that when we detect a stimulus omission, the brain may automatically switch mechanisms between temporal grouping and temporal prediction, depending on the SOAs.

## Results

### Reaction time for the missing oddball depends on the SOA

**Figure 1:**
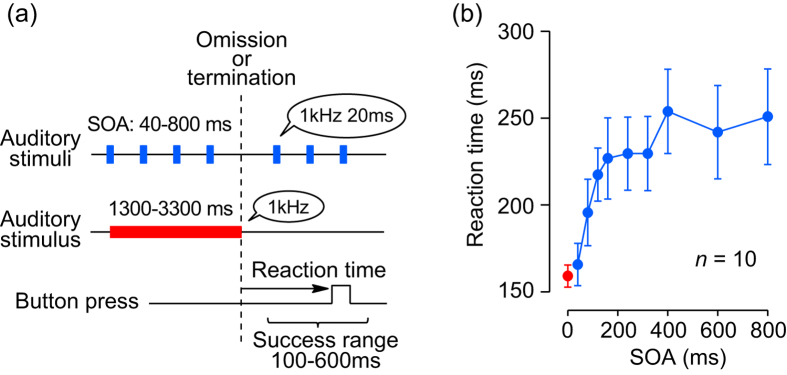
Relationship between reaction time and SOA (Experiment 1). (**a**) Subjects were asked to press a button in response to a single omission of repetitive auditory stimuli (upper) or the termination of continuous sound (middle), as quickly as possible. The SOA ranged from 40–800 ms, and each repetitive stimulus lasted 20 ms. (**b**) Reaction time for different SOAs. Data for the continuous sound are shown at zero SOA (red dot). Error bars indicate 95% confidence intervals. Note that the reaction time strongly depended on the SOA (one-way ANOVA, F_(8, 81)_ = 6.33, *p* < 0.001).

In Experiment 1, ten subjects were asked to press a button as soon as they detected a single omission of isochronously repetitive auditory stimuli (Fig. 1a; Supplementary Audio S1 and S2). Subjects almost always responded to the stimulus omission within 100–600 ms (range: 92–100%, mean ± SD: 98.3 ± 2.8%, *n* = 10). Figure 1b plots reaction time as a function of SOA, showing a significant effect of SOA (one-way ANOVA, F_(8, 81)_ = 6.33, *p *< 0.001). In trials with shorter SOAs (<250 ms), reaction time depended strongly on the SOA, whereas it was almost constant in trials with longer SOAs. This was demonstrated statistically through a linear regression analysis for the data of individual subjects (shorter SOAs: slope = 0.31, Pearson’s *r* = 0.57, *df* = 48, *p* < 0.001; longer SOAs: slope = 0.02, *r* = 0.12, *df* = 38, *p* = 0.48).

While the timing of upcoming stimuli can be reliably predicted when we listen to a stimulus sequence with longer SOA (Supplementary Audio S2), this is impossible for a sequence with shorter SOA. Instead, the train of discrete sounds with a short SOA can be perceptually grouped and regarded as a single continuous stream (Supplementary Audio S1). Based on this perceptual impression, we hypothesized that for shorter SOAs the brain temporally integrates sensory signals for each stimulus and monitors the transient reduction of neuronal activity from the steady state to detect the stimulus omission (Supplementary Fig. S1a). To test this possibility, trials with a long-lasting continuous sound (1 kHz, random 1300–3300 ms, Supplementary Audio S3) were interleaved randomly with the missing oddball trials, and reaction time to the termination of the sound was measured. The data (Fig. 1b, red dot) were in good agreement with the relationship between the reaction time and the SOA, under the assumption that the continuous sound had zero onset asynchrony.

### Detection of stimulus omission for shorter SOAs relies on temporal grouping

Since neurons in auditory cortex are known to exhibit sustained activity in response to a long-lasting pure-tone stimulus^11^, detecting the termination of a continuous sound is likely to depend on the decay of sustained activity following the stimulus offset (Supplementary Fig. S1a, top row). If a single omission of repetitive sounds with short SOAs is also detected by monitoring for the brief reduction in neuronal activity, its detectability must be strongly affected by the baseline fluctuation of steady state neuronal activity (Supplementary Fig. S1a, middle rows). To achieve temporal integration of discrete sounds, the same population of neurons may need to be activated by each stimulus in the sequence>^12^. Otherwise, the high fluctuation of baseline activity would preclude the detection of the omission-induced decay in neuronal activity (Supplementary Fig. S1b).

**Figure 2:**
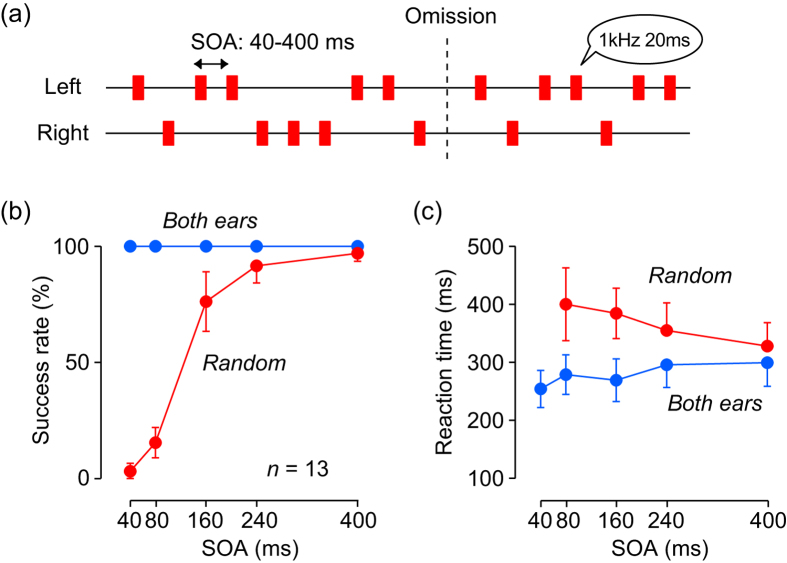
Random monaural condition (Experiment 2). (**a**) Each repetitive stimulus was randomly presented to either ear with a constant SOA (40–400 ms). Subjects were asked to detect the stimulus omission correctly but not in hurry. (**b**) Proportions of correct trials in the random monaural (red dots) and binaural (blue) conditions. (**c**) Reaction time for different SOAs in both conditions. Error bars indicate 95% confidence intervals.

To test these possibilities, we examined the performance under conditions in which the repetitive stimuli randomly activated different populations of neurons. In Experiment 2, each stimulus in the sequence was monaurally presented to a randomly chosen ear, with a constant SOA (Fig. 2a; Supplementary Audio S4 and S5). Under this condition, detection of stimulus omission was difficult for shorter SOAs and was virtually impossible in trials with 40-ms SOA (3.1 ± 6.3%, *n* = 13, Fig. 2b), even though the subjects were asked to detect the stimulus omission as accurately as possible. In contrast, they could reliably detect the stimulus absence when SOA was 400 ms in the monaural condition (96.9 ± 6.3%), or when each stimulus was presented binaurally (100% for all SOAs, Fig. 2b, blue dots). A two-way ANOVA showed significant main effects (stimulus condition, F_(1, 120)_ = 635, *p* < 0.001; SOA, F_(4, 120)_ = 132, *p*  <  0.001) and interaction (F_(4, 120)_ = 132, *p* < 0.001) for the rate of successful trials. Post hoc multiple comparisons revealed significant differences between stimulus conditions for SOAs of 40–160 ms (*t*-test with Bonferroni correction, *p* < 0.01/5) but not for SOAs of 240 and 400 ms (*p* = 0.04 and 0.10, respectively, which were greater than the corrected alpha value of 0.05/5). Even in successful trials, reaction times were longer for shorter SOAs in the random ear (monaural) condition compared with the both ear (binaural) condition (*t*-test with Bonferroni correction for SOAs of 80 and 160 ms, *p* < 0.01/5, Fig. 2c). These results suggest that the temporal integration mechanism for stimulus sequence might underlie oddball detection only for trials with shorter SOAs.

**Figure 3:**
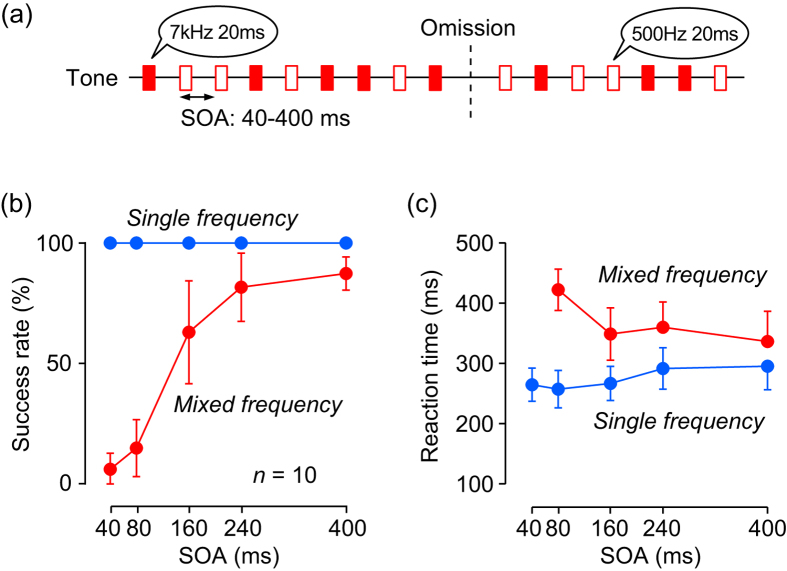
Mixed-frequency condition (Experiment 3). (**a**) Each stimulus was randomly chosen from two different pitches (7 kHz or 500 Hz pure tones). (**b,c**) Conventions are the same as in Fig. 2 except that red and blue dots indicate the data for the mixed- and single-frequency conditions, respectively.

Because of the tonotopic organization of the central auditory pathways^13^, different frequencies are also expected to activate different populations of neurons. In Experiment 3, each of isochronously repetitive auditory stimuli was chosen randomly from two different frequencies (0.5 or 7 kHz, Fig. 3a; Supplementary Audio S6 and S7). For the control (single frequency) condition, either 0.5 or 7 kHz tone was presented in each trial. The data for the two tones were combined because no significant difference was detected (two-way ANOVA, F_(1, 90)_ = 0.02 and 1.19 for the success rate and reaction time, respectively, *p* = 0.89 and 0.28). The results of the mixed frequency condition were quite similar to Experiment 2. For shorter SOAs, detection of stimulus omission was difficult (Fig. 3b) and reaction times were longer (Fig. 3c). A two-way ANOVA showed significant main effects (stimulus condition, F_(1, 90)_ = 264, *p* < 0.001; SOA, F_(4, 90)_ = 31, *p* < 0.001) and interaction (F_(4, 90)_ = 31, *p* < 0.001) for the rate of success trials. Post hoc multiple comparisons revealed significant differences between stimulus conditions for all but 240-ms SOAs (*t*-test with Bonferroni correction, *p* < 0.05/5). In terms of reaction time, multiple comparisons detected significant differences between conditions for SOAs of 80 and 160 ms (*p* < 0.05/5) but not for the other SOAs. These results suggest that the auditory system might be unable to integrate randomly-alternating discrete sounds into a single continuous stream even when interstimulus interval is short.

### Dual processing interferes with oddball detection for longer SOAs

In Experiments 2 and 3, we found the stimulus conditions under which the detection of stimulus omission was impaired only when SOAs were short. We next explored conditions that disrupted oddball detection specifically when SOAs were long. Since highly demanding tasks require more cognitive resources^14^,^15^, we expected that simultaneous prediction of two different events might be limited by cognitive capacity.

**Figure 4:**
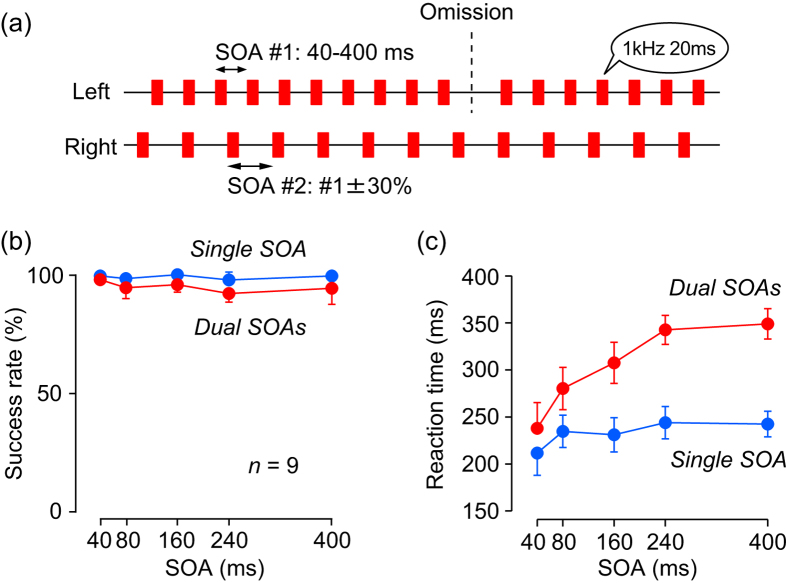
Dual-task condition (Experiment 4). (**a**) Repetitive stimuli with different SOAs were presented simultaneously to each ear. Stimulus omission occurred randomly in either ear. (**b,c**) Conventions are the same as in Fig. 2 except that red and blue dots indicate the data for the dual- and single-SOA conditions, respectively.

In Experiment 4, we generated two series of isochronous auditory stimuli (1 kHz, 20 ms) that differed in SOA, and simultaneously presented one to each ear (the dual-SOA condition). In each trial, the stimulus omission occurred in only one randomly selected ear (Fig. 4a; Supplementary Audio S8 and S9) so that the subjects needed to keep track of both rhythms simultaneously. In general, the subjects could reliably detect the absence of a regular beat under both the dual (Fig. 4b, red dots) and single (blue dots) conditions. A two-way ANOVA revealed a small but significant effect of stimulus condition (dual vs. single, F_(1, 80)_ = 14.01, *p* < 0.001) on the rate of successful trials, but no effect of SOA (F_(4, 80)_ = 1.38, *p* = 0.24) or the interaction between them (F_(4, 80)_ = 0.43, *p* = 0.78). In contrast, reaction time differed greatly between conditions, particularly for longer SOAs (Fig. 4c). A two-way ANOVA detected significant main effects of stimulus condition and SOA on latency (stimulus condition, F_(1, 80)_ = 123.42, *p* < 0.001; SOA, F_(4, 80)_ = 16.71, *p* < 0.001), as well as the interaction between them (F_(4, 80)_ = 5.78, *p* < 0.001). Compared with the control (single SOA) condition, reaction times across subjects for longer SOAs (≥ 240 ms) were greatly prolonged (range: 62–136 ms, mean ± SD: 103 ± 26 ms, *n* = 9) in the dual SOA condition, while those for shorter SOAs were only slightly delayed (31–84 ms, 50 ± 15 ms). A one-way ANOVA showed that the difference in mean reaction time between conditions significantly depended on SOA (F_(4, 40)_ = 18.61, *p* < 0.001). Thus, dual temporal processing highlights a second mechanism underlying oddball detection that might be automatically chosen when SOAs are long.

### Detection of stimulus omission in other sensory modalities

**Figure 5:**
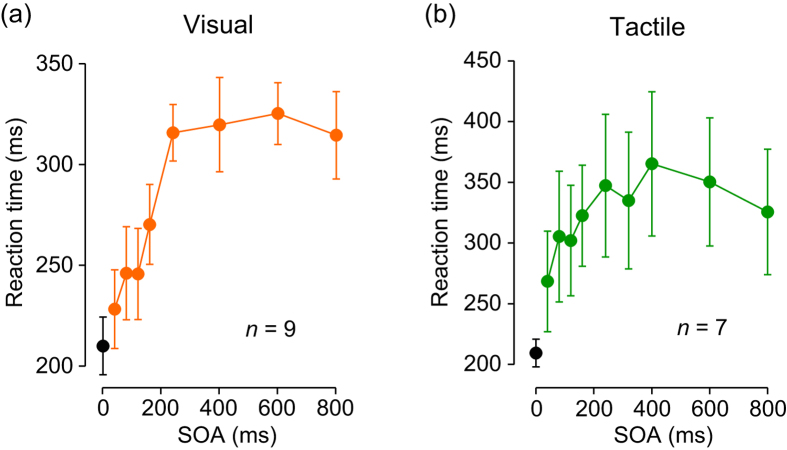
Different modalities (Experiment 5). Relationship between reaction time and SOA for repetitive visual (**a**) and tactile (**b**) stimuli. Subjects were asked to respond as quickly as possible, and the trials with premature response (<100 ms) were repeated in a block. Error bars indicate 95% confidence intervals.

We next examined whether the two detection mechanisms could be generalized to other sensory modalities. In Experiment 5, the subjects were asked to detect a stimulus omission in a series of discrete visual or tactile stimuli, as quickly as possible. When we repeatedly flashed LEDs (20 ms), the timing of each next stimulus could be reliably predicted for the sequence of a 400-ms SOA (Supplementary Movie S2), but not for a 40-ms SOA (Supplementary Movie S1), similarly to the impression to a series of auditory stimuli. The relationship between reaction time and SOA significantly altered depending on the SOA (one-way ANOVA, F_(7, 64)_ = 14.97, *p* < 0.001, Fig. 5a). For shorter SOAs (<250 ms), the reaction time significantly depended on SOA (regression slope = 0.42, Pearson’s *r* = 0.70, *df* = 43, *p* < 0.001), whereas it was almost constant for longer SOAs (slope = −0.013, *r* = −0.07, *df* = 25, *p* = 0.72). Furthermore, reaction time to the offset of persistent illumination (Fig. 5a, black dot) was well predicted by the regression line for the shorter SOAs. Similar results were obtained when the subjects were presented with a series of tactile stimuli (Fig. 5b). Again, the reaction time altered for different SOAs (one-way ANOVA, F_(8, 54)_ = 2.23, *p* = 0.039, Fig. 5b). The reaction time strongly depended on SOA for shorter SOAs (40–240 ms; regression slope = 0.36, *r* = 0.37, *df* = 33, *p* < 0.05), but did not for longer SOAs (slope = −0.04, *r* = −0.10, *df* = 26, *p* = 0.60). Taken together with the data from Experiment 1 (Fig. 1b), these results suggest that two different neuronal mechanisms might underlie the detection of stimulus omission in oddball paradigms across different sensory modalities.

## Discussion

We found that the detection of stimulus omission in a sequence of isochronous auditory stimuli differed depending on the SOA. For SOAs shorter than 250 ms, the integration of signals from different neural populations appeared to be impossible (Experiments 2 and 3). In contrast, for longer SOAs, the subjects had a difficulty in keeping track of two tempos presented simultaneously to different ears (Experiment 4). This double dissociation of impaired detection in the missing oddball task suggests that the brain may automatically select from two alternative neuronal mechanisms depending on the SOA.

For shorter SOAs, series of isochronous repetitive sounds might be grouped into a single continuous auditory stream, allowing subjects to detect the stimulus omission as a brief cessation of the stream. In auditory scene analysis, sounds with different features are segregated from each other while those with similar properties are grouped together and streamed over time^2^,^16^,^17^. This ‘auditory stream segregation’ appears to be useful when discriminating sounds from different sources.

Two neural mechanisms have been proposed for stream segregation^12^. The temporal coherence theory indicates that stimuli simultaneously activating different population of neurons are perceptually combined^18^. However, because no temporally-coherent stimuli were presented in our experiments, this theory cannot explain the present results. In contrast, the population separation theory indicates that sounds activating the same population of neurons are grouped together^12^,^19^. This theory could account for our findings that auditory streaming was generated only when each sound in the sequence likely activated the same population of neurons. Schematic drawings in Supplementary Fig. S1a illustrate how the auditory system may detect stimulus omission. If the SOA is short enough, responses to each stimulus might be temporally integrated within slow-adapting neurons, and baseline fluctuation would be relatively small (second row, blue band). Stimulus omission could be detected by monitoring the brief reduction in neuronal activity that surpasses the baseline fluctuation. However, if the SOA is longer, baseline fluctuation becomes greater, and the signal-to-noise ratio decreases (third row), making detection more difficult and resulting in longer reaction time. Thus, the model can account for the specific dependence of reaction time on SOA in shorter range that was found in this study (Figs 1b and 5). When the SOA is much longer, temporal integration would no longer occur, and another mechanism, such as temporal prediction, must be used to detect stimulus omission.

Because a similar relationship between reaction time and the SOA was also found for visual and tactile repetitive stimuli (Fig. 5), the two detection mechanisms might also function for different sensory modalities. Similarly to the sound sequence, the timing of each next light flash can be reliably predicted when SOA is long (Supplementary Movie S2), while the repetitive flash can be perceptually grouped when SOA is short (Supplementary Movie S1). In addition, the previous study showed that a series of tactile stimuli with short SOAs can be streamed over time only when stimuli were applied to the same location^12^. Thus, a similar streaming mechanism may also exist for other sensory modalities.

For SOAs longer than 240 ms, reaction time was relatively constant and was consistently shorter than the SOA (Fig. 1a), indicating that sensory feedback from the following auditory stimuli played no role in the detection of stimulus absence. Considering the temporal limitation of auditory streaming^2^, it is unlikely that the brain generates sustained activity for periodic sounds with SOAs >250 ms (Supplementary Fig. S1a, bottom row). Instead, because we can consciously predict timing of each stimulus when SOAs are longer (e.g., Supplementary Audio S2), the brain might generate prediction error signals when a regularly presented stimulus is absent.

In support of this hypothesis, our subjects were able to detect stimulus omission during random stimulus presentation at 400-ms SOA (Experiments 2 and 3), indicating that the brain could integrate signals from different ears or tonotopic maps. Although how the signals from different neuronal populations are integrated remains unknown, one possibility is that only timing information (or ‘time markers’) are extracted, and then used to acquire the rhythms and predict the timing of upcoming stimuli. For the perceptual binding of different sensory attributes or modalities, a relatively slow temporal limit of 2–3 Hz has been reported^1^, which corresponds to an SOA of 167–250 ms. This system for extracting, integrating, and monitoring time markers from different neuronal populations might be recruited when we are attempting to detect stimulus omission for longer SOAs.

Our results also showed that although all subjects could detect stimulus omission when two series of repetitive stimuli with different SOAs were presented simultaneously to each ear (Fig. 4b), the reaction times for longer SOAs were greatly prolonged, while those for shorter SOAs were only slightly delayed (Fig. 4c). This was in stark contrast to the greater deficits associated with shorter SOAs in Experiments 2 and 3. The reduced performance in the dual-task paradigm suggests that the detection of stimulus omission for longer SOAs may rely on cognitive processes requiring attention and working memory. Because the prediction of upcoming stimulus timing inevitably involves the memory of stimulus intervals and monitoring of time from the preceding stimulus, these findings are consistent with the notion that when SOAs are longer, the brain computes prediction error signals for the absence of regular stimulus.

Although our results of double dissociation strongly suggest two different mechanisms for the detection of stimulus omission, the different properties could be explained by bistability of a single non-linear system^20^,^21^. For example, the phase transition of syncopated tapping and bimanual rhythmic coordination patterns have been explained by single non-linear models^20^. In addition, rhythm and pitch can be viewed as a continuum along the frequency and the perceptions of the two could be described using a non-linear model^22^. Nevertheless, the actual neural mechanisms and/or sites for rhythm and pitch may be different^23^. Building such a theoretical model for different properties in the missing oddball paradigm is far beyond the scope of the present study.

Relevant to our behavioural paradigms, event-related potentials to the omission of repetitive stimuli has been extensively examined^24^,^25^,^26^,^27^,^28^,^29^,^30^,^31^. In support of our hypothesis of two different mechanisms, the properties of the omitted stimulus potential (OSP) differ depending on the SOA^6^. For the sequence of visual stimuli, the ‘fast’ OSP is generated for the omission of light flashes of 5–40 Hz (SOA of 25–200 ms), while the ‘slow’ OSP is generated for flashes of <1.6 Hz (SOA of >625 ms)^6^. The fast OSP requires stable fixation but not attention, and is observable even in non-mammal vertebrates^32^. Interestingly, these properties are in accord with our ‘single-population’ hypothesis of temporal grouping for shorter SOAs, which might be operated rather automatically and free from higher-order cognitive processes. In contrast, the slow OSP requires the intention to detect stimulus omission and exhibits binocular or multisensory interactions^6^. Similarly, for the auditory stimuli, the fast OSP can be evoked for stimulus sequence of 1–20 Hz (SOA of 50–1000 ms) without attention, while the slow OSP is generated for the train of <4 Hz (SOA of >250 ms) when the subjects attend to the stimuli^33^. The previous studies have shown that the slow OSP precedes the detection of stimulus termination in the stop-reaction time task^10^ and the slope and magnitude of slow OSP correlates with the reaction time^34^. Taken together with our present findings, these results suggest that when the SOA is sufficiently long, the slow OPS may play a causal role in the detection of stimulus omission, although the neural mechanism for the generation of slow OPS remains elusive.

A number of studies suggest that the cerebro-cerebellar networks are implicated in rhythm processing irrespective of motor involvement^35^,^36^,^37^. Recent studies suggest that the beta-band coherence between the cerebello-thalamo-cortical pathways might transmit temporal information of rhythmic stimuli, reflecting the prediction of upcoming stimulus timing^38^. Similarly, our previous study in non-human primates showed neuronal modulation in the cerebellum that was proportional to the SOA^7^. Because inactivation of the cerebellar dentate nucleus delayed the detection of stimulus omission only for longer SOAs, the cerebellum might play a role in temporal prediction but not in perceptual streaming. Additional studies are needed to address how the signals from the cerebellum are converted into the prediction error signals for stimulus omission that ultimately trigger movements.

In summary, we found several different properties in detecting a single omission of isochronous repetitive sounds, depending on the SOA. Our results suggest that two distinct mechanisms might be selected automatically when detecting stimulus omission. For shorter SOAs the detection of stimulus omission may rely on perceptual streaming of discrete sounds, while for longer SOAs temporal prediction of each upcoming stimulus may be necessary. Because a similar change in reaction time was observed for periodic visual and tactile stimuli, automatic switching between these two mechanisms may be generalized to other sensory modalities. The transition of these strategies might be determined by the threshold for temporal prediction, and may involve many cortical and subcortical structures.

## Methods

### Subjects

Nineteen healthy individuals (18–43 years old), including both authors, participated in this study. Because we did not intend to compare the data across experiments, the data for different experiments were collected from different but overlapped subject populations. Ten subjects participated in more than 3 experiments, while five subjects participated in only one experiment. All had normal hearing and normal or corrected-to-normal vision, and provided written informed consent in advance of the experiments. Experimental procedures described below were evaluated and approved by the Ethics Committee of Hokkaido University Graduate School of Medicine, and all experiments were performed in accordance with the guideline of the same committee. Except for the authors, all participants were naïve to the purpose of the experiments when we collected the data. No subject was excluded from data analysis. Post-hoc analyses without the data from the authors showed qualitatively similar results to those summarized in figures, and all critical statistical tests described in the Results still showed significant differences even without the data obtained from the authors.

### Apparatus and stimuli

In all experiments, subjects sat on a chair in front of a desk. They were asked to press a button on the desk using the right index finger to control the task (see below). In Experiments 1–4, auditory stimuli (0.5, 1 or 7 kHz; 20 ms in duration) were generated by function generators (FG-274, TEXIO), and presented through headphones (HP-AL102, Victor; 65 dB SPL measured at the ear pad by presenting continuous sound). In Experiment 5, three blue light-emitted diodes (LEDs) mounted on a black board were illuminated as visual stimuli, which were placed 40 cm from the subject. Tactile stimuli were presented to the left index finger using a hand-made vibrator that was made from an audio speaker (MM-SPL2N, Sanwa Supply). To eliminate sounds, the vibratory film was removed, and the subjects touched the vibration coil that was driven by a 250-Hz square-wave signal (SEN-8203, Nihon Kohden). During tactile stimulation (Experiment 5), ear plugs were used and masking white noise (80 dB SPL) was presented via headphones (SE-M290, Pioneer). The behavioural task and data acquisition were controlled by the TEMPO system (Reflective Computing). Experimental events were updated every 5 ms and the data were sampled at 1 kHz.

### Experimental paradigms

In all experiments, each trial started when subjects pressed the button and terminated when they responded to the stimulus omission by pressing the same button. The instruction was given for each subject by presenting a document that was read aloud by the experimenter.

In Experiment 1, 500 ms following the initial button press, either a series of repetitive auditory stimuli or a continuous sound (both 1 kHz) was presented (Fig. 1a; Supplementary Audio S1–3). In trials with repetitive stimuli, a 20-ms tone was repeated with a fixed SOA and a single tone was omitted abruptly (‘missing oddball’ paradigm). The SOA was chosen pseudo-randomly for each trial from the set of 40, 80, 120, 160, 240, 320, 400, 600, or 800 ms. The omission occurred randomly during the interval ranging from 2400–8000 ms (2400–4000 ms for SOAs ≤240 ms, 3200–4800 ms for SOAs ≤400 ms, 4200–6000 ms for SOA of 600 ms, and 5600–8000 ms for SOA of 800 ms) following the first stimulus. Subjects were asked to respond to the omission by pressing the button. In trials with continuous sound, subjects were also asked to respond immediately to the termination of the sound. The termination occurred randomly during the interval ranging from 1300–3300 ms. Each session contained ten trials for each SOA (100 trials in total). Different trial types were randomly interleaved in a block. To examine the reaction time, the subjects were asked to press a button as soon as possible without minding premature response. When reaction time was shorter than 100 ms or longer than 600 ms, the trial was treated as an error, and the trial was subsequently repeated in the block. Incorrect performance was informed by a 50-Hz tone (700 ms). Because the preliminary experiments showed that naïve subjects sometimes exhibited a delayed response, data for analysis were collected only after three training sessions. Supplementary Fig. S2 shows the effects of training on the reaction time for 5 subjects.

In Experiment 2, each stimulus (1 kHz, 20 ms) in the sequence was presented monaurally to randomly-chosen ear while the SOA remained constant in each trial (random ear condition, Fig. 2a; Supplementary Audio S4 and S5). This stimulus condition was presented in half of the trials, while all stimuli were presented binaurally (both ear condition) in the remaining trials. In both conditions, the SOA ranged from 40 to 400 ms (40, 80, 160, 240, or 400 ms). To examine the detectability of stimulus omission, the subjects were asked to respond carefully rather than quickly in Experiment 2. Failed trials were not repeated. Thus, reaction time was measured under different instruction from Experiment 1. For each stimulus condition, the mean reaction time was computed only when the proportion of successful trials was >20%. Data for analysis were obtained after one training session.

In Experiment 3, each stimulus in the series was randomly chosen from two tones with different frequencies (7-kHz or 500-Hz tones, different-frequency condition). This stimulus condition was presented in half of the trials (Fig. 3a; Supplementary Audio S6 and S7), while in the remaining trials a single tone (either 7-kHz or 500-Hz) was presented in each trial (single-frequency condition). Again, the subjects were asked to detect stimulus omission carefully rather than quickly, and the failed trials were never repeated. Data were collected after one training session.

In Experiment 4, two different series of isochronous repetitive stimuli (1 kHz, 20 ms, different SOAs) were presented to different ears (dual-SOA condition, Fig. 4a; Supplementary Audio S8 and S9). This stimulus condition was presented in half of the trials, while in the remaining trials a single sequence was presented to both ears (single-SOA condition). In both conditions, a stimulus omission occurred in only one ear, which was chosen randomly in each trial. For the sequence with the omission, the SOA was selected from the set of 40, 80, 160, 240, or 400 ms. In the dual SOA condition, the SOA for the other side (without omission) was 0.7- or 1.3-times relative to the selected SOA. Subjects were asked to press a button as quickly as possible without minding any premature response. Because the detection of stimulus omission in the dual-SOA condition was somewhat difficult especially for longer SOAs, data were collected only after two training sessions.

In Experiment 5, we repeated Experiment 1 in different sensory modalities (Fig. 5). The visual stimulus was a series of 20-ms LED flashes (Supplementary Movies S1 and S2) and the tactile stimulus was a series of 20-ms vibrations at 250 Hz. As in Experiment 1, the subjects were instructed to press the button as quickly as possible, and the failed trials including premature responses were repeated later in a block. For both sensory modalities, responses within 100–700 ms of stimulus omission were considered as correct detection. Data were collected after 2 training sessions for each modality.

### Data analyses

Data were saved in file during experiments and were analysed offline using Matlab. For each trial, reaction time was measured from the time of stimulus omission. The effects of stimulus conditions on the reaction time and the rate of successful trials were assessed using a two-way analysis of variance (ANOVA). Post hoc multiple comparisons (*t*-test with Bonferroni correction) were also conducted as necessary. For Experiments 1 and 5, the effects of SOA on reaction time were evaluated firstly by one-way ANOVA and then separately examined for short and long SOAs using a linear regression analysis applied to the means of individual subjects. Details of other statistical measures are reported in the relevant text. Because the data for different experiments were obtained from different subject populations, all statistical comparisons were made within each experiment.

To simulate how individual neurons can temporally integrate discrete signals, we assumed model neurons that responded rapidly to a brief auditory stimulus and exhibited a decaying activity with a long time constant (Supplementary Fig. S1). The time course of neuronal activity was taken from the previous study that simulated the decay of auditory sensation^39^. Briefly, the transient activity had double exponential time course with the rising phase with a 60-ms time constant and the decaying phase with a 160-ms time constant. The time courses of neuronal activity were simulated for the trains of 20-ms stimulus pulses with SOAs of 40, 200, and 400 ms (Supplementary Fig. S1).

## Additional Information

**How to cite this article**: Ohmae, S. and Tanaka, M. Two different mechanisms for the detection of stimulus omission. *Sci. Rep.* **6**, 20615; doi: 10.1038/srep20615 (2016).

## Supplementary Material

Supplementary Information

Supplementary Audio S1

Supplementary Audio S2

Supplementary Audio S3

Supplementary Audio S4

Supplementary Audio S5

Supplementary Audio S6

Supplementary Audio S7

Supplementary Audio S8

Supplementary Audio S9

Supplementary Movie S1

Supplementary Movie S2
