# Supplementary material for: Two different mechanisms for the detection of stimulus omission
Source: Sci Rep. 2016 Feb 5;6:20615. doi: 10.1038/srep20615 (PMC4742881; doi:10.1038/srep20615)
Supplement: Supplementary Information [file srep20615-s1.pdf]

Supplementary Information

“Two different mechanisms for the detection of stimulus omission”

by Shogo Ohmae & Masaki Tanaka

Supplementary Figures: 2
Supplementary Audio Clips: 8
Supplementary Movies: 2

Supplementary Figure S1 for Ohmae & Tanaka

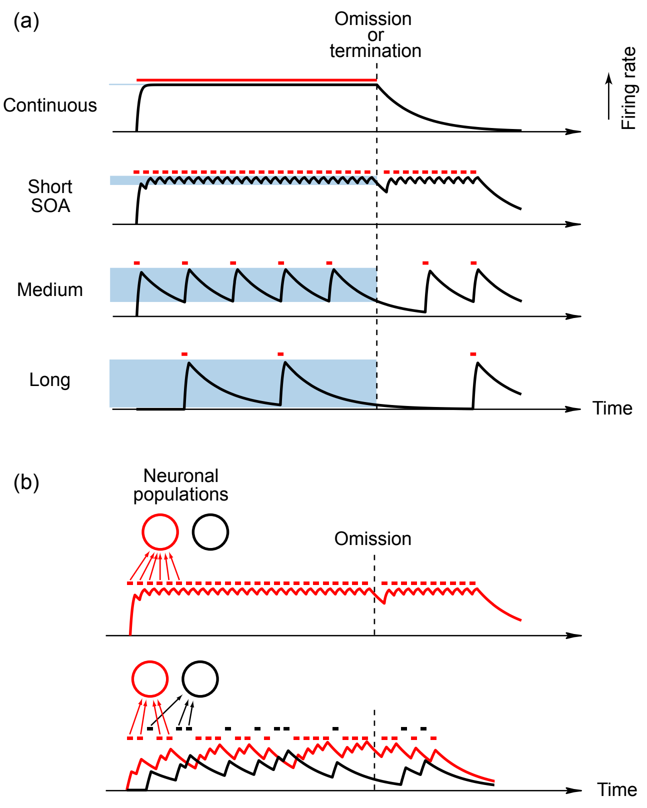

Supplementary Figure S1 | Possible detection mechanisms. (a) Activity of a hypothetical slow-adapting neuron during Experiment 1. Timings of auditory stimuli are shown as red bars. Black traces indicate neural activity for continuous sound (top) and a series of discrete sounds with different SOAs (bottom rows). The dashed line indicates the termination or omission of the stimulus. Because neuronal activity decays slowly, responses to successive stimuli temporally overlap for the sequence with a short SOA. In the missing oddball paradigm, subjects can rely on detection of a brief reduction of neuronal activity to report the stimulus omission or termination. In this case, the baseline fluctuation of sustained neuronal activity (blue shadow) can be viewed as the noise for the detection of the signals. Note that longer SOAs are associated with greater noise, which may cause longer reaction times. At the longest SOA (bottom), temporal integration for successive stimuli does not occur, implying that another mechanism, such as temporal prediction, is necessary for the detection of stimulus omission. (b) Temporal integration can be achieved only when a single ensemble of neurons responds equally to individual stimuli in the sequence (top, as in Experiment 1). Even when SOAs are sufficiently short, the sustained activity in each neuron becomes unstable if individual stimuli randomly excite different neuronal populations (bottom, as in Experiments 2 and 3). In both panels, red and black circles indicate different neuronal populations responding to specific auditory stimuli (denoted by bars and arrows in different colours).

Supplementary Figure S2 for Ohmae & Tanaka

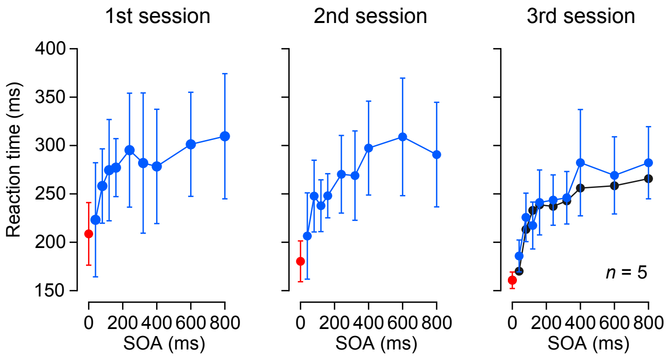

Supplementary Figure S2 | Effects of training in Experiment 1. Reaction time gradually decreased during the three training sessions. For comparison, the data for the analysis (4th session) are shown as black circles in the right panel. Conventions are the same as in Figure 1b. In these plots, data from only 5 naïve subjects are shown because the remaining 5 subjects including the authors did not perform all the stimulus conditions during the training sessions and also participated in the preliminary data collection before the experiments.

Captions for Supplementary Audio Clips

Supplementary Audio S1 | Experiment 1, SOA = 40 ms.

Supplementary Audio S2 | Experiment 1, SOA = 400 ms.

Supplementary Audio S3 | Experiment 1, continuous stimulus.

Supplementary Audio S4 | Experiment 2, random monaural stimulus, SOA = 40 ms.

Supplementary Audio S5 | Experiment 2, random monaural stimulus, SOA = 400 ms.

Supplementary Audio S6 | Experiment 3, mixed-frequency condition, SOA = 40 ms.

Supplementary Audio S7 | Experiment 3, mixed-frequency condition, SOA = 400 ms.

Supplementary Audio S8 | Experiment 4, dual-SOA condition, SOAs = 40 and 52 ms.

Supplementary Audio S9 | Experiment 4, dual-SOA condition, SOAs = 400 and 520 ms.

Captions for Supplementary Movies

Supplementary Movie S1 | Experiment 5, light flashes, SOA = 40 ms.

Supplementary Movie S2 | Experiment 5, light flashes, SOA = 400 ms.
